# Expectations of health care quality among rural Maya villagers in Sololá Department, Guatemala: a qualitative analysis

**DOI:** 10.1186/s12939-017-0547-5

**Published:** 2017-03-14

**Authors:** Matthew Ippolito, Anita Chary, Michael Daniel, Joaquin Barnoya, Anne Monroe, Michelle Eakin

**Affiliations:** 1 Department of Medicine, Johns Hopkins University School of Medicine, 1830 E Monument St Rm 450-B, Baltimore, MD 21287 USA; 2 Department of Anthropology, Washington University in St. Louis, 1 Brookings Drive, Campus Box 1114, St. Louis, MO 63130 USA; 3 Johns Hopkins University School of Medicine, 1000 Eager Street, Baltimore, MD 21202 USA; 4 Department of Surgery, Washington University in St. Louis, 660 S. Euclid Avenue, Campus Box 8100, St. Louis, MO 63110 USA; 5 Department of Medicine, Johns Hopkins University School of Medicine, 1830 E Monument St Rm 8060, Baltimore, MD 21287 USA; 6 Department of Medicine, Johns Hopkins University School of Medicine, 5501 Hopkins Bayview Circle, Baltimore, MD 21224 USA

**Keywords:** Latin America, Guatemala, Maya, Kaqchikel, Public health, Health care utilization, Qualitative study

## Abstract

**Background:**

Indigenous populations in Latin America have worse health outcomes than their nonindigenous counterparts. Differences in access to and use of biomedical resources may explain some of the observed disparities. Efforts to address these differences could be aided in part by better understanding the socio-medical contexts in which they occur.

**Methods:**

We performed a qualitative analysis of field notes collected during a 2008 program evaluation of a health post in a rural Maya village in Sololá Department, Guatemala. Forty-one interviews were conducted among a community-based convenience sample of adult men and women. Interviews focused on experiences, perceptions, and behaviors related to the local biomedical and ethnomedical health care resources.

**Results:**

Penetrance of the local health post was high, with most (90%) of respondents having accessed it within the prior five years. The prevailing attitude toward the health post was positive. We identified facilitators and barriers to health post use that corresponded with three thematic areas: clinic operations, visits and consultations, and medical resources. Proximity to the home, free consultations and medications, and social support services were among the most commonly cited facilitators. Barriers included limited clinic hours, medication stock-outs, provision of care that did not meet patient expectations, and unavailability of diagnostic tests.

**Conclusions:**

In a rural Maya community in Guatemala, operational and quality-based factors, independent of sociocultural considerations, informed the perception of and decision to access biomedical resources. Interventions that address these factors may increase health care utilization and alleviate some of the health disparities that accompany indigeneity in Guatemala and similar contexts.

## Background

Latin America is home to indigenous groups that fare worse than their nonindigenous counterparts by several socioeconomic determinants: there are large gaps in earnings, educational achievement, life expectancy, and maternal-child health outcomes [1]. Among the Latin American countries, Guatemala has the second largest proportion of indigenous people. Approximately 50% of its population identify as belonging to one of 21 distinct indigenous groups, which include Maya, Xinca, and Garifuna peoples [2]. Life expectancy countrywide for Maya peoples, who account for the majority of the indigenous population in Guatemala, is 13 years lower than among the non-indigenous population, rates of maternal mortality are up to four times higher, and childhood stunting is 50% more prevalent [3]. Efforts to address these health disparities could be helped in part by elucidation of the sociomedical contexts in which they occur.

In indigenous communities, Western biomedicine-based health services often exist alongside a parallel network of ethnomedical healing systems that include midwifery, herbalism, and shamanism [4]. Which services are accessed, who accesses them, and how the personal decision is made to seek care are among the factors that may affect community health and wellbeing [5]. Biomedical health services are underutilized worldwide, and in Latin America even more so than other low- and middle-income countries [6].

In Guatemala, the constitution guarantees free government-sponsored health care to all citizens. The Guatemalan Ministry of Health (MOH) offers care through a three-tiered public health system, which includes health posts providing basic primary care and vaccinations in rural villages, health centers providing basic primary care and some emergency services in towns and small cities, and hospitals providing specialized care and emergency services in urban areas and department capitals. While this three-tiered system is designed to offer health care coverage to approximately 70% of the population, understaffing and resource shortfalls limit the availability of services as well as actual population coverage [4]. Public health services in Guatemala are accessed less frequently by those below the poverty line compared to those above [3, 4, 6]. While the factors contributing to health disparities between the indigenous Maya population and non-indigenous are multiple and complex, some of the disparity may result from underutilization of health services among the former [1, 6–8].

**Fig. 1:**
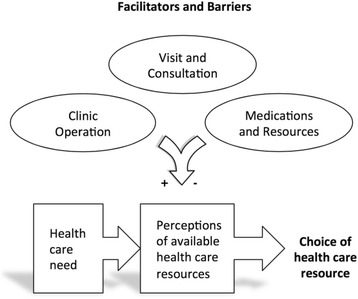
Conceptual framework of the influence of facilitators and barriers on community members’ perceptions of available health care resources and impact on health care utilization. Adapted from Levesque et al. [17]

To date, most studies examining health care-seeking attitudes and behaviors among rural Maya people in Guatemala focus on sociocultural barriers to care, and suggest that underutilization of biomedical health services occurs due to unmet cultural needs and resistance to outside influence [8–15]. Additionally, these studies largely focused on maternal-child health. The role of potential barriers such as perceived quality of care, demand for services, and ready access to high-quality secondary and tertiary care is less frequently explored [4, 16]. In this broader context, we performed a qualitative investigation of lay utilization of a health post run in a rural Maya village in the Sololá Department of Guatemala. Our study aimed to elucidate factors that inform community members’ decision to use or not use locally available biomedical services for visitation and consultation when health care needs arise (Fig. 1) [17].

## Methods

A qualitative study was performed in the context of a 2008 program evaluation of a local health post (*puesto de salud*) in a rural Guatemalan village located in the Sololá Department. The health post is operated collaboratively by the MOH and a non-governmental organization (NGO) founded and developed by health care professionals from the United States. In this paper, we use the pseudonym Santa María to refer to this village in order to protect the anonymity of research participants.

### Study site

Santa María is a rural Kaqchikel Maya community of approximately 1,200 people. It is notable for its relative isolation, being accessible only by ferryboat or footpath. The local economy is predominantly agrarian. Most residents are involved in raising coffee for export and vegetables for local sale, supplemented by subsistence farming of corn and beans. The literacy rate is 28%, compared to the national average of 82%, and average weekly family income is US$20 [9]. Most residents speak the Mayan language Kaqchikel, and some are bilingual in Kaqchikel and Spanish. The village health post is located centrally and is co-administered by the Guatemalan MOH and a United States-originating aid organization. Providers include one Spanish-speaking physician from the United States, one Spanish-speaking Guatemalan physician, and two local Spanish- and Kaqchikel-speaking midlevel practitioners (nurse-clinicians). In addition to health services, the clinic offers social assistance programs, cooking and nutrition classes, and other educational activities. Villagers also receive care from independent midwives and two health promoters (*promotores de salud*). The health promoters are community health workers who operate out of their homes providing care for minor ailments. Traditional healers include herbalists (*curanderos*) and bone doctors in nearby villages, and local Maya spiritual leaders (*ajq’ija’*). Several kiosks and small shops in the village sell traditional herbs as well as biomedical pharmaceutical medicines. The nearby town of Panajachel (population: 11,142) has private clinics and pharmacies, which are also accessed by the villagers, and the closest hospital is in the department capital, Sololá (population: 30,155).

### Study population

The study population was a convenience sample of female and male villagers in homes and workplaces and included both health post users and non-users. Inclusion criteria included age ≥18 years, ability to participate in an interview, current residence in the village, and provision of verbal informed consent. Using purposeful sampling, the team recruited interview participants through home and workplace visits conducted throughout the entire village from July to August 2008 at various times, including evenings and weekends.

### Data collection

We developed a semi-structured interview guide consisting of open- and closed-ended questions as a tool to evaluate perceptions and attitudes pertaining to the health post and health services utilization. Interviews focused on each participant’s pattern of accessing the health post and other biomedical resources, use of traditional ethnomedical resources, and perceived advantages and disadvantages of using services at the health post and elsewhere. During interviews, follow-up questions were asked to probe new topics that emerged. Throughout data collection, the interview guide was iteratively modified to focus on predominant topic areas from preceding interviews. Socio-demographic characteristics including age, gender, occupation, and language fluency were also collected.

The survey team consisted of the first author and a translator. The translator was a bilingual Spanish- and Kaqchikel-speaking resident of Santa María. Interviews were conducted in Kaqchikel, Spanish, or a combination based on interviewee preference. Participant responses and the interviewers’ observations were recorded in detailed paper-based field notes.

### Analytic approach

Data were analyzed stepwise using a structured grounded theory approach to identify themes relating to expectations of health care quality and utilization of biomedical resources [18]. MI manually reviewed transcribed field reports for assignment of unique codes using thematic analysis [19]. The reports were manually reviewed a second time by MI with application of the codes to specific sections and phrases. Codes were then organized into three core categories. After open coding was completed, the core categories were evaluated and refined by four of the authors (MI, MD, AM, and ME) each of whom had also reviewed the primary reports. All data were collated and analyzed using Excel 14.6 (Microsoft, Seattle, WA).

### Ethical considerations

The study was deemed exempt by the Johns Hopkins University School of Medicine Institutional Review Board. Ethical approval of the 2008 program evaluation was waived by the Committee for the Protection of Human Rights of the Geisel School of Medicine at Dartmouth College. All study participants were verbally informed in the indigenous language of study aims and confidentiality of responses, and provided verbal consent. The study was performed with the consent and knowledge of local representatives of the health post and NGO.

## Results

Forty-one semi-structured interviews were completed among 21 men and 20 women ranging in age from 23 to 78 years (median age: 40 years). Additional demographic features of the respondents are described in Table 1. Interviews lasted on average 30 min with a range of 10 to 60 min. All but four-three men and one woman-had accessed the health post at some point in the preceding five years. Acute rather than chronic conditions predominated; the most common were musculoskeletal pain and gastrointestinal illness. Three themes emerged from the analysis. Participants conveyed attitudes, observations and behaviors pertaining to (1) clinic operations, (2) visits and consultations, and (3) medications and other medical resources. Table 2 outlines these themes and the specific facilitators and barriers identified from respondents’ narratives.

**Table 1:** Socio-demographic characteristics of participants

Characteristic	Women	Men
No. participants	20	21
Age, years, median (range)	41 (23–78)	38 (23–77)
Language use, %		
Spanish and Kaqchikel	14	94
Kaqchikel only	86	6
Occupation, no.		
Construction worker	0	8
Homemaker	11	0
Farmer	0	2
Fisherman	0	2
Weaver	6	0
Other	2^a^	7^b^
Retired	1	1

^a^Midwife and hotel worker

^b^Driver, ferryman, librarian, merchant, and tailor

**Table 2:** Factors influencing community members’ perceptions of a rural health post in Sololá Department, Guatemala

Core category	Influential Factor	
	Facilitator	Barrier
Clinic operations	Health post is located close to home.	Health post hours of operation are unclear.
	Consultation time with provider is quick and convenient.	Health post hours of operation are limited.
		Consultation time with provider is too long.
		Health post wait time is too long.
		Participant is immobile or home-bound and health post does not provide home visits.
Visits and consultations	Participant prefers physician over midlevel provider.	Provider does not speak participant’s preferred language.
	Professionalism is displayed by staff and providers.	Participant harbors mistrust toward provider.
		Health post clerical staff interaction is negative.
		Participant prefers injection over orally administered medications.
		Provider does not perform physical examination.
Medications and resources	Free medications are provided by the health post.	Health post is unable to fill medication prescription from own stock.
	Social assistance programs are sponsored by the health post.	Health post distributes expired medications.
	Educational and training activities are offered by the health post.	X-ray and other diagnostic tests are not available.

### Convenience of access and clinic operations

Participants most readily identified the clinic’s proximity to their homes and cost-free access to services as the two key features promoting use. Interviewees cited their ability to walk to the village center to access services at the post, as opposed to having to pay transportation costs for a ferry ride to facilities located outside of the village. Interviewees also viewed free health care as beneficial given their limited income.

Notably, interviewees qualified their otherwise favorable perception of the free services and medications provided by the health post with statements about the perceived quality of care. According to some respondents, private clinics in other towns offered superior services to the local health post, and they attributed this difference at least in part to the fact that private clinics charged for their services. Fees for service were viewed as an incentive for private practitioners and their staff to “attend well” to patients, and foster a “business”-like environment. Their impression was that because private clinics charged for their services, they were motivated to treat their patients professionally. Private clinics were also perceived as better able to maintain a more exhaustive supply of medications and diagnostic equipment than their public counterparts.

*‘If you go to the National Hospital [in Sololá], they do not attend well [to the patients], but with the private doctors it is more like a business …. Private doctors are much better.’* 26-year-old man.

*‘The people go to the [private] doctors in Panajachel and Sololá because the clinic [in the village] lacks equipment.’* 33-year-old man.

Other operational factors that were mentioned related to health post hours, waiting times, and visit duration. Lack of transparency of hours of operation and limited business hours were detractions for interviewees. Some participants commented that health post hours were not clear; they reported episodes of visiting the health post during usual opening hours and finding the health post closed or the providers absent. One respondent’s perception of the clinic was that it was “always closed”:

*‘The health post is always closed. The health post’s hours are a problem.’* 35-year-old woman.

Interviewees, and particularly male interviewees, identified the health post’s limited hours of operation as a deterrent to care seeking. Men in the community appeared to access the health post less frequently than women, after accounting for clinic visits by women seeking care for their children rather than themselves. Among participants who reported limited health post hours as a barrier to access, five out of seven were men. The men in the village tended to work in the surrounding forests and fields, or traveled to the markets in nearby towns to sell and trade goods, rendering them absent from the village during regular health post hours. Long wait times were a deterrent, and while some respondents lauded short, efficient consultation times, others desired more time with providers than was allotted in a given visit.

### Provider training, shared language, and expectations of care provision and professionalism

Interviews prominently featured comments and observations about clinic visits and clinical consultations. These included preferences regarding providers’ level of training, language, and approaches to care, as well as opinions about encounters with support staff. Interviewees expressed expectations for a professional demeanor among clinic receptionists, and there were rare expressions of patient-provider mistrust.

Gender preference was not mentioned, but preferences were stated for provider level-of-training. Among those who reported a preference, physicians were preferred over nurse-clinicians; physicians were viewed as having more experience and more medical expertise than non-physician providers. There was also a preference for Kaqchikel-speaking providers, indicated more frequently by women than men, who overall were statistically significantly less likely to use Spanish compared to the men (Table 1).

Reasons for seeking care elsewhere than the health post included accessibility, perceived quality of care, and availability of diagnostic tests. Men were more likely to choose to visit a health promoter over the health post due to evening and weekend accessibility of the village’s two health promoters. Private doctors were visited either after a consultation at the health post did not result in the desired outcome, such as medication not being prescribed or provided, or when there was a perceived need for a diagnostic test such as a radiograph, unavailable at the health post.

Personal health beliefs about perceived efficacy of medication influenced choice of services. We identified a belief held by some that injection medicines, and medicines administered directly to the affected area such as creams or drops, were more effective than those given by the oral route. Some participants preferentially sought care from health promoters who had a reputation for administering injections, and one mother whose child had an eye infection traveled to town to purchase eye drops at a pharmacy because she was unsatisfied with the pills given her by the health post that same day.

Most interviewees expected that a visit to the doctor would include physical examination. For example, one participant voiced concern over going to the clinic for stomachache and diarrhea and not receiving a physical examination. Some villagers noted which providers were more likely to examine them and which were not, and requested their preferred provider accordingly.

Patient-provider relationships were generally described in very positive terms, with two instances of respondents conveying mistrust. In one case, mistrust stemmed from the death of a respondent’s grandchild years prior who had been evaluated at the health post and sent home where the child died shortly thereafter. In the second case, a woman gave birth prematurely and felt that she had been misinformed by the clinic providers regarding her due date.

*‘They brought my 1-year-old grandchild to the clinic because he had fevers and was coughing, and they saw him and gave some medicines, but he died the next day…. The nurses committed a crime.’* 72-year-old woman.

*‘[I was told] the child would come on the nineteenth of November but the child came on the fifteenth of October.’* 28-year-old woman.

Clinic support staff at the health post were reported by some respondents to have sometimes shown a disrespectful attitude (“*mala cara*”*)*. A few respondents related episodes of miscommunication, such as being told by a receptionist that a provider was not available when the patient was previously told by the provider herself that she was available for consultation. One respondent suggested that professionalism among clerical staff might be improved if pay was increased:

*‘The health post should pay the receptionists more so that they treat the patients better.’* 45-year-old man.

### Free medications and other resource considerations

The third theme that emerged from interviews centered on the cost, availability, and quality of health care resources. These included comments and observations about medications-provided for free by the health post-and those related to the ease or difficulty of obtaining follow-up tests and procedures.

Free medications were an important draw to the health post among the villagers, who generally had low-earning jobs (Table 1) and often lived below the poverty line [9]. Participants reported medication stock-outs, which are known to be a frequent occurrence in health posts due to factors such as underfunding and logistical challenges [20, 21]. In addition to stock-outs, there were concerns among some respondents who had been dispensed expired medications.

*‘Free medicine is the most important thing the clinic offers.’* 35-year-old woman.

*‘There are not enough of them [medications].’* 42-year-old man.

*‘When my mother went to the health post, the health post had no medications for her.’* 23-year-old man.

The lack of an X-ray machine in the village compelled some to forgo even initial evaluation at the health post and travel outside of the village to seek care for orthopedic complaints. Other clinic offerings such as cooking and nutrition classes, social assistance programs, and educational and training activities were popular among respondents who had used or were aware of them.

## Discussion

In a qualitative analysis of 41 interviews, we found that community members of a rural Maya village in Sololá Department, Guatemala exhibit quality-based health care attitudes that may impact their decision to access care. We identified influential factors among three core categories: clinic operations, visits and consultations, and medications and resources. Our study expands beyond populations typically reached in prior studies by recruiting a non-clinic based sample that included men, and whose health concerns were not limited to maternal-child healthcare [7–12].

The health post had a very high penetrance, with nearly every participant relating a clinic visit within the last five years. When accessing biomedical services, the participants described informed expectations and needs they want met. Judgments were based on accessibility of the clinic and providers, professionalism among clerical staff, provider training, provision of care, and availability of medications and diagnostic tests. Our findings are consistent with attitudes and expectations for high quality care encountered by investigators in other low- and middle-income countries [22–24]. Increased awareness of other health care contexts through media and other channels, expanded availability of biomedical resources, and government and international investments offer potential explanations for these findings [25].

Prior studies of health-seeking behaviors among Maya people in Guatemala focused predominantly on sociocultural barriers [8–15]. In contrast, our study supports the notion that Maya people in rural, resource-poor settings harbor practical expectations for the biomedical care they receive and seek out health care platforms that meet these expectations. Sociocultural considerations, while manifested in terms of some participants’ therapeutic expectations, were less emphasized in interviews than provider training and communication, reliable access to resources, and professionalism. Villagers expressed a preference for knowledgeable providers who are able to communicate in a shared language, which has also been described in other health care settings in Guatemala [26]. They emphasized wishes for a sufficiently stocked pharmacy with pre-expiration date medications. Interviewees described expectations of professionalism wherein support staff are courteous and clinic hours transparent and convenient. Our finding of perceived associations of privatized, fee-based care with professional, quality care above that of the free government option has also been reported in the context of diabetes and mental health in Guatemala [4].

There is an extensive literature on feelings of mistrust among indigenous groups towards health systems [13–16, 27–31], and while our study revealed elements of it, mistrust was not a prominent theme. Unlike prior studies [29], none of our participants reported denial of care due to language or cultural barriers. Previous studies also describe episodes of perceived mistreatment on account of ethnic differences [16]. While participants in our study related episodes of mistreatment and other negative experiences, they did not attribute these episodes to prejudicial attitudes but rather to lack of professionalism, training, or errors in clinical management.

There are limitations to this qualitative study. First, the sample was not drawn at random, which reduces the generalizability of our findings, although a range of experiences were captured among both men and women of varying ages. Second, the presence of an NGO collaborator distinguishes the Santa María health post from other local health posts in Guatemala, which tend to be solely administered by the MOH and may not possess a similar extent of resources, number of providers, or level of provider training. Third, the presence of a community outsider may have constrained candid discussion of certain topics and introduced reporting bias. Finally, data are from responses collected in 2008, and changes in demographics, clinic operations, and the government health system’s local funding allocation during the intervening time period may reduce the applicability of our results.

Overall, this study contributes actionable insights into an understudied high-risk population. Fostering a culture of professionalism among clinic staff might be achieved through training, formalization of clinic procedures, and adopting a mission statement exemplifying patient-centered care. Adaptive systems to decrease clinic wait times might take advantage of mobile technology that is now nearly ubiquitous in rural Guatemala, already successfully harnessed to improve health care delivery in other low- and middle-income settings [32]. Health posts administered by the Guatemalan MOH are occasionally closed for weeks at a time as providers attend training and travel on holiday, or as staff turnover occurs; publicizing clinic hours through accurate signage and advance notification of planned closures could ease frustration on the part of care seekers. While often arising from central budget shortfalls and logistic factors otherwise beyond the control of local health posts, the specter of medication stock-outs might be mitigated by central supply chain analyses and strict inventory, tools that have been deployed with success in sub-Saharan Africa [33]. Ensuring the availability and proficiency of medical translators when providers are unable to communicate in the indigenous language could narrow linguistic and cultural gaps that contribute to patient dissatisfaction, such as when the treatment offered is not the treatment expected [34].

## Conclusion

Indigeneity remains a risk factor both for poverty and poor health outcomes in Guatemala and other contexts. Knowledge of indigenous groups’ experiences and expectations is needed to optimize programs that aim to address disparities. Health care provision at the local level should receive the same quality-based considerations in low- and middle-income countries as in high-income countries.

## Abbreviations

MOH: Ministry of health
NGO: Non-governmental organization
